# Cervicovaginal microbiota significantly changed for HPV-positive women with high-grade squamous intraepithelial lesion

**DOI:** 10.3389/fcimb.2022.973875

**Published:** 2022-08-04

**Authors:** Chunlei Guo, Wenkui Dai, Qian Zhou, Liming Gui, Han Cai, Di Wu, Jun Hou, Changzhong Li, Shuaicheng Li, Hui Du, Ruifang Wu

**Affiliations:** ^1^ Department of Obstetrics and Gynecology, Peking University Shenzhen Hospital, Shenzhen, China; ^2^ Institute of Obstetrics and Gynecology, Shenzhen Peking University-Hong Kong University of Science and Technology Medical Center (PKU-HKUST) Medical Center, Shenzhen, China; ^3^ Department of Obstetrics and Gynecology, Peking University Shenzhen Hospital, Shenzhen Key Laboratory on Technology for Early Diagnosis of Major Gynecologic Diseases, Shenzhen, China; ^4^ Department of Computer Science, City University of Hong Kong, Hong Kong, Hong Kong SAR, China

**Keywords:** cervicovaginal microbiota, Chinese women, HPV infection, low squamous intraepithelial lesion, high squamous intraepithelial lesion

## Abstract

Lower female genital tract is colonized by a variety of microbes (cervicovaginal microbiota, CVM) which associate with the risk of genital infection. This study characterized CVM for 149 Chinese women with different status of human papillomavirus (HPV) infection and squamous intraepithelial lesion (SIL): no HPV infection (HPV-), HPV infection without significant SIL (HPV+NoSIL), HPV infection with low-grade SIL (HPV+LSIL) and HPV infection with high-grade SIL (HPV+HSIL). Analysis results showed CVM has dramatically changed in HPV+HSIL group when compared to HPV+LSIL group, but it exhibited no significant differences between HPV- and HPV+NoSIL groups as well as between HPV+NoSIL and HPV+LSIL groups. In consistence, random forest analysis found more notable differences in HPV+HSIL vs HPV+LSIL comparison than in other comparisons. In addition, depletion of *Lactobacillus* in CVM was more to be frequently identified in SIL-positive women as compared to SIL-negative individuals. Our findings suggested that significant CVM differences occurred when SIL developed to HSIL which was caused by persistent HPV infection.

## Introduction

Persistent human papillomavirus (HPV) infection is the highest risk of invasive cervical cancer (ICC) (Hildesheim et al., 2007; The FUTURE II Study Group, 2007). Given the limited prophylactic vaccine inoculation, HPV infection is impacting a large number of women in developing countries (Hildesheim et al., 2007; The FUTURE II Study Group, 2007). Emerging studies found that cervicovaginal microbiota (CVM) associated with clinical outcomes of HPV infection, that is, HPV clearance, regression from squamous intraepithelial lesion (SIL), persistent HPV infection or progression to pre-cancerous intraepithelial neoplasia and even ICC (Brotman et al., 2014; Reimers et al., 2016; Di Paola et al., 2017; Brusselaers et al., 2019; Berggrund et al., 2020; Dareng et al., 2020; Mitra et al., 2020; Tamarelle et al., 2019; Usyk et al., 2020).

CVM with dominated *Lactobacillus* is prevalent in healthy women who had no genital infections (Ravel et al., 2011; Gajer et al., 2012; Chaban et al., 2014; Chen et al., 2017; Ma et al., 2020). A number of studies identified depletion of *Lactobacillus* and increased bacterial diversity for CVM in women with HPV infection (Gao et al., 2013; Lee et al., 2013; Mitra et al., 2015; Oh et al., 2015; Seo et al., 2016; Zhang et al., 2018; Zhou et al., 2019; Borgogna et al., 2020; Cheng et al., 2020; So et al., 2020). Nevertheless, some researchers found no marked differences between HPV-positive and HPV-negative women (Reimers et al., 2016; Chao et al., 2019; Nieves-Ramírez et al., 2021). Additional studies found that CVM structures changed significantly in women with SIL, as compared to that in HPV-negative and HPV-positive women (Gao et al., 2013; Mitra et al., 2015; Oh et al., 2015; Seo et al., 2016; Zhang et al., 2018; Borgogna et al., 2020; So et al., 2020; Nieves-Ramírez et al., 2021). In addition, CVM with distinct dominant *Lactobacillus* species represented different clinical outcomes for HPV-positive women (Gao et al., 2013; Oh et al., 2015; Seo et al., 2016; Di Paola et al., 2017; Zhang et al., 2018; Wang et al., 2019; Mitra et al., 2020; Norenhag et al., 2020; So et al., 2020; Usyk et al., 2020). For instance, women with *Lactobacillus crispatus*-dominated CVM were positively associated with natural clearance of pre-existing HPV infection, but the risk of persistent HPV infection increased for other CVM structures (Wang et al., 2019; Mitra et al., 2020; Norenhag et al., 2020; Usyk et al., 2020). The impact of CVM on clinical outcomes of HPV infection could be attributed to the association of immune responses with CVM (Doerflinger et al., 2014; Ojala et al., 2014; Anahtar et al., 2015; Łaniewski et al., 2018; Ilhan et al., 2019; Łaniewski et al., 2019; Al-Nasiry et al., 2020; Łaniewski et al., 2020), which could be impacted by ethnicity and lifestyles (Zhou et al., 2007; Ravel et al., 2011; Gajer et al., 2012; Noyes et al., 2018). Though several reports had analyzed the CVM differences between HPV-negative and HPV-positive (with or without SIL) women in China (Chen et al., 2019; Chen et al., 2020; Wu et al., 2020; Zhang et al., 2021), there was lack of discussion for the association of CVM with HPV infection and different levels of SIL for Chinese women.

This study included 149 Chinese women who met the inclusion criteria and had different status of HPV infection and SIL. We aimed to understand the CVM differences between HPV- (HPV-negative and no significant SIL), HPV+NoSIL (HPV-positive but no significant SIL), HPV+LSIL (HPV-positive and low-grade SIL) and HPV+HSIL (HPV-positive and high-grade SIL) groups, and to interpret the association of CVM with HPV infection as well as SIL level. Our findings should provide extensive insights into the correlation between CVM and HPV infection for Chinese women.

## Materials and methods

### Ethics approval

This study was approved by the Ethics Committee of Peking University Shenzhen Hospital (registration number: 2021-006).

### Sample collection, HPV testing, cytological and pathological diagnosis

Women older than 18 years and sex-exposed were eligible to be enrolled. Exclusion criteria were as follows: pregnant or lactation; post-menopause; with a history of cervical ablation or resection surgery or hysterectomy or pelvic radiotherapy; suffering from autoimmune diseases or HIV infection; taking immunosuppressants, oral antibiotics, vaginal douching; having sex one week before colposcopy; having positive results for H_2_O_2_, leukocyte esterase, neuraminidase, β-glucuronidase or acetylaminoglucosidase (detected by the bPR2014A platform, Jiangsu Bioperfectus Technologies Co., Ltd.). The vaginal swabs were preserved in a 2 ml sterile tube and stored at -80 centigrade within 30 minutes after collection for sequencing in six months.

Cervical exfoliated cells were obtained by cervical brushes and were preserved in 20ml SurePath^®^ Preservative Solution. Then the Cobas 4800 platform (Roche Molecular Diagnostics, Pleasanton, CA.), which is approved by FDA for HPV testing, was applied to detect 14 high-risk HPV genotypes *via* collected cell samples: HPV16, HPV18 or other 12 high-risk HPV genotypes (HPV 31/33/35/39/45/51/52/56/58/59/66/68). For pathological diagnosis, we used directed biopsies for all visible lesions, and random biopsies at the squamocolumnar junction in normal quadrants. The highest grade among the multiple biopsies from each quadrant was recorded as the final diagnosis. Pathological diagnosis were reported as negative, cervical intraepithelial neoplasia (CIN) grade I, II, III, or cancer. According to the criteria of the Bethesda system, CIN I and CIN II/III were classified as LSIL and HSIL respectively.

### Microbial DNA extraction, 16S rRNA gene amplicon sequencing and data processing

Microbial DNA was extracted by Dneasy PowerSoil Pro Kit (Qiagen, German). The DNA concentration and purity were estimated by 1% agarose gels on Agilent5400 (Agilent Technologies, Inc., Santa Clara, USA). Then we amplified 16S rDNA V4-V5 hypervariable regions (515-FR: GTGCCAGCMGCCGCGGTAA, 926-RR: CCGTCAATTCMTTTRAGTTT) of 16S rRNA gene and determined the quality of PCR products (Qubit, Thermo Fisher Scientific, Singapore). Qualified DNA libraries were then sequenced by 250bp read length based on Illumina NovaSeq platform (Illumina, San Diego, CA, United States).

### Bioinformatics analysis

To estimate the sample size for each group, we conducted power analysis by the R *pwr* package. In this study, we randomly enrolled the patients with/without HPV infection, and furtherly divided the patients into four groups. The power and significant one-way level w set as 0.8 and 0.05 respectively, and the large ANOVA effect size was 0.4. Then the sample size was estimated to be >= 24 for each group. Given inter-individual variations for vaginal microbiota, we enrolled more than 24 patients in each group. Raw sequencing data were filtered and annotated as previously reported (Chen et al., 2017). Wilcoxon rank-sum test was applied to analyze inter-group differences of diversity, bray-curtis dissimilarity, CVM structures, and the p value was adjusted by the Benjamini & Hochberg method. The impact of HPV infection, SIL and age on CVM distributions was assessed by Permutational Multivariate Analysis of Variance (PERMANOVA) with 9,999 permutations (package “vegan” in R). All microbial samples were clustered based on jensen-shannon distance. Random forest classifier (100 trees, five-fold cross-validation) was utilized to assess the importance of bacterial species in inter-group differences (package “mlr” in R). Analysis results were visualized using R software (version 4.1.2).

## Results

### Overview of sample information

A total of 149 Chinese women aged from 19 to 50 years were enrolled in this study, of whom 30 were HPV negative (defined as Group HPV- averaged 38.5(29-49) years old), 40 were HPV-positive and had no significant SIL (defined as Group HPV+NoSIL, averaged 37.7(22-50) years old), 28 were HPV-positive and pathological diagnosed with LSIL (defined as Group HPV+LSIL, averaged 33.5(21-47) years old), and 51 were HPV-positive and pathological diagnosed with high-grade HSIL (defined as Group HPV+HSIL, averaged 32.1(19-47) years old). For SIL-negative and -positive women who was HPV-positive, HPV16 infection (including mixed infection with other HPV genotypes) represented 12.9% and 44.3% respectively, HPV18 infection (including mixed infection with other HPV genotypes) represented 5.7% and 11.4% respectively, and the other 12 high-risk HPV infection accounted for 47.1% and 59.5% respectively. All recruited women did not smoke, and had no menopause as well as genital infections other than HPV.

### CVM differed between HPV-, HPV+NoSIL, HPV+LSIL and HPV+HSIL group

**Figure 1:**
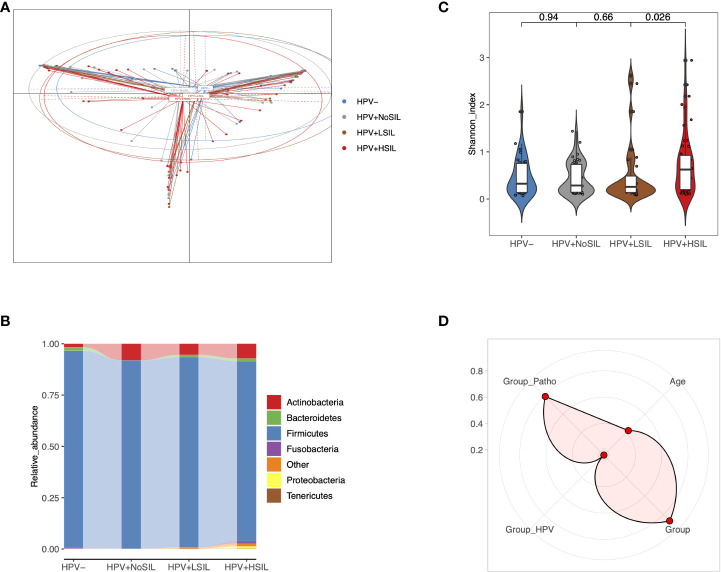
Overall CVM differences between HPV-, HPV+NoSIL, HPV+LSIL and HPV+HSIL group. **(A)** Principal coordinate analysis (PCoA) based on Jensen-shannon divergence as the distance measurement. **(B)** Comparison of alpha-diversity as HPV infection and SIL developed *via* Shannon index. **(C)** Distribution of phylum-level CVM components across four groups. **(D)** Permutational multivariate analysis of variance (PERMANOVA) to assess the effects of several indices on CVM structure: more significant if the point is located near the outer circle. HPV-: women without HPV infection and significant SIL; HPV+NoSIL: women with HPV infection but without significant SIL; HPV+LSIL: women with HPV infection and LSIL; HPV+HSIL: women with HPV infection and HSIL. Group_Patho: levels of SIL, including no significant SIL (NoSIL), LSIL, HSIL; Group_HPV: negative and positive; Group: HPV-, HPV+NoSIL, HPV+LSIL and HPV+HSIL. Wilcoxon rank-sum test was applied to analyze the statistical significance for inter-group differences.

Principal coordinate analysis (PCoA) indicated slight differences of microbial samples between HPV-, HPV+NoSIL, HPV+LSIL and HPV+HSIL group (**Figure 1A**). Further analysis showed critically elevated bacterial diversity for HPV+HSIL group when compared to HPV+LSIL group (**Figure 1B**). By contrast, there were no significant differences between HPV+LSIL and HPV+NoSIL, as well as between HPV+NoSIL and HPV- group (**Figure 1B**). In consistence, phylum-level analysis found increased diversity of taxonomic phylum in HPV+HSIL group when compared to other three groups, including elevated levels of Fusobacteria, Proteobacteria and Tenericutes (**Figure 1C**). In addition, permutational multivariate analysis of variance (PERMANOVA) suggested that SIL severity had obviously higher contribution to CVM differences, as compared to HPV infection (**Figure 1D**).

### HPV+HSIL group had significant genus-level differences when compared to HPV-, HPV+NoSIL and HPV+LSIL group

**Figure 2:**
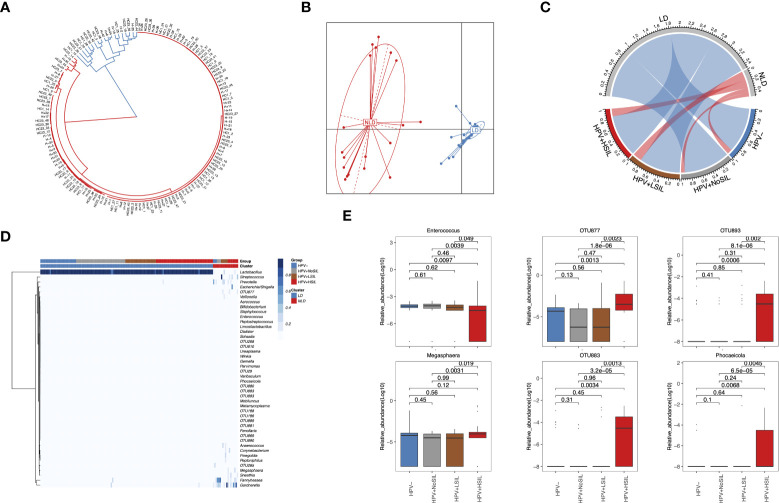
Genus-level CVM differences between HPV-, HPV+NoSIL, HPV+LSIL and HPV+HSIL group. **(A)** Clustering of all microbial samples. **(B)** PCoA based on Jensen-shannon divergence as the measurement of distance for LD and NLD groups. **(C)** Distribution of LD and NLD CVM structures across four groups. The color of lines in the circle represents different CVM structures: blue, LD; red, NLD. The width of lines represent proportion of specific CVM structure in the group. **(D)** Genus-level CVM components for different groups and CVM clusters (LD and NLD). **(E)** Several genera which had significant changes in HPV+HSIL group as compared to other groups. HPV-: women without HPV infection and significant SIL; HPV+NoSIL: women with HPV infection but without significant SIL; HPV+LSIL: women with HPV infection and LSIL; HPV+HSIL: women with HPV infection and HSIL. Wilcoxon rank-sum test was applied to analyze the statistical significance for inter-group differences.

At genus level, all microbial samples could be clustered to *Lactobacillus*-dominated (LD) and non-*Lactobacillus*-dominated (NLD) CVM respectively (**Figures 2A, B**). Further analysis showed higher proportion of microbial samples which had NLD CVM in women with SIL (10%, 7.5%, 17.86% and 15.69% for HPV-, HPV+NoSIL, HPV+LSIL and HPV+HSIL respectively) (**Figure 2C**). Accumulated bacterial genera in NLD CVM mainly were *Gardnerella* (median(IQR), 17.89(0.1, 30.09)% vs 0.03(0.01, 0.06)% in LD CVM), *Fannyhessea* (0.1(0.05, 23.67)% vs 0.02(0.009, 0.03)% in LD CVM), *Megasphaera* (0.01(0.003, 2.56)% vs 0.006(0, 0.009)% in LD CVM), *Prevotella* (4.3(0.14, 14.18)% vs 0.003(0, 0.01)% in LD CVM), *Sneathia* (0.009(0, 0.08)% vs 0(0, 0.003)% in LD CVM), and *Streptococcus* (0.11(0.03, 4.30)% vs 0.05(0.02, 0.06)% in LD CVM) (**Figure 2D**). Nevertheless, the relative abundance of *Lactobacillus* in the CVM represented insignificant differences between four groups (**Figure 2D**). In HPV+HSIL group, the level of *Enterococcus* decreased significantly, whereas *Megasphaera* and several unclassified open taxonomy units (OTUs) signally accumulated, including OTU877, OTU883 and OTU893 (**Figure 2E**). Though the relative abundance was low, *Phocaeicola* had higher relative abundance in HPV+HSIL group as compared to other groups (**Figure 2E**). However, microbial samples in HPV+LSIL, HPV+NoSIL and HPV- group had insignificant inter-group differences at genus level (**Figures 2D, E**).

### The different importance of bacterial species in HPV- vs HPV+NoSIL, HPV+NoSIL vs HPV+LSIL and HPV+LSIL vs HPV+HSIL comparison

**Figure 3:**
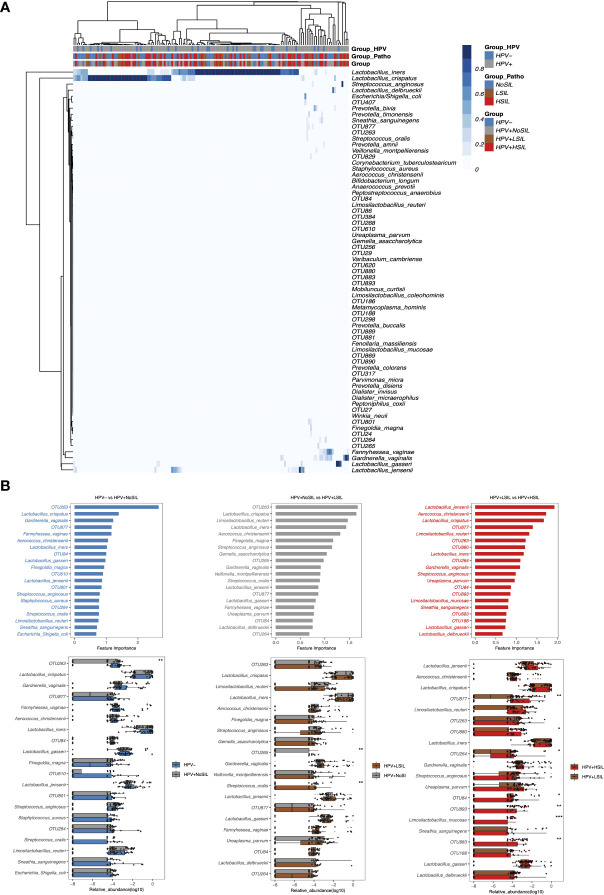
Random-forest analysis of species-level CVM differences between HPV-, HPV+NoSIL, HPV+LSIL and HPV+HSIL group. **(A)** Species-level CVM components for different groups (Group: HPV-, HPV+NoSIL, HPV+LSIL and HPV+HSIL; Group_Patho: no significant SIL (NoSIL), LSIL and HSIL; Group_HPV: negative and positive). **(B)** Comparison of microbial samples between one group and another group which had higher severity of HPV infection and SIL, like HPV- vs HPV+NoSIL. The upper three figures represented the importance of top 20 bacterial species to differentiate two groups, and the importance decreased from top to down. The lower three figures represented inter-group differences for the top 20 genus. HPV-: women without HPV infection and significant SIL; HPV+NoSIL: women with HPV infection but without significant SIL; HPV+LSIL: women with HPV infection and LSIL; HPV+HSIL: women with HPV infection and HSIL. Wilcoxon rank-sum test was applied to analyze the statistical significance for inter-group differences. p-value: *, **, *** and **** represent <0.05, <0.01, <0.001 and <0.0001 respectively.

At species level, all microbial samples were clustered to several CSTs which were dominated by *L. crispatus*, *Lactobacillus. iners*, *Lactobacillus. gasseri*, *Lactobacillus. delbrueckii*, several *Lactobacillus* species and non-*Lactobacillus* bacterial species respectively (**Figure 3A**). And women with SIL had higher levels of non-*Lactobacillus* CVM than women in HPV- and HPV+NoSIL groups (**Figure 3A**). Additionally, random forest analysis found different importances and components for the top 20 bacterial species which contributed to inter-group differences (**Figure 3B**). For HPV- vs HPV+NoSIL and HPV+NoSIL vs HPV+LSIL comparison, we identified 15 common bacterial species and OTUs in the top 20 important features (**Figure 3B**). OTU263, which was classified as *Streptococcus*, ranked the first in differing HPV+NoSIL from HPV- and HPV+LSIL from HPV+NoSIL group (**Figure 3B**). Further analysis found that only OTU265 and *Streptococcus oralis* had striking differences between HPV+NoSIL and HPV+LSIL group, and OTU263 had significant difference between HPV- and HPV+NoSIL group (**Figure 3B**). Nonetheless, the comparison between HPV+HSIL and HPV+LSIL group indicated more differences as compared to the aforementioned comparisons, including the remarkable accumulation of OUT877, OTU880, OTU264, OTU893 and OTU883 in CVM for HPV+HSIL group (**Figure 3B**). There were no significant differences of *Lactobacillus* species when HPV infection occurred or SIL progressed (**Figure 3B**).

## Discussion

A growing number of studies demonstrated the association of CVM with HPV infection. However, HPV acquisition was mainly impacted by sex activities and thus possible for women with any CVM structure (Bruni et al., 2010; Dai et al., 2021), suggestive of heterogeneous CVM for HPV-positive women (Gao et al., 2013; Chao et al., 2019). In addition, CVM structure can be impacted by many factors such as hygiene habits, ethnicity and age. This may explain inconsistent findings for CVM analysis in different cross-sectional studies (Gao et al., 2013; Lee et al., 2013; Mitra et al., 2015; Oh et al., 2015; Reimers et al., 2016; Seo et al., 2016; Zhang et al., 2018; Chao et al., 2019; Zhou et al., 2019; Borgogna et al., 2020; Cheng et al., 2020; So et al., 2020; Nieves-Ramírez et al., 2021). Though HPV infection can orchestrate immune responses and then affect CVM structures, VM-host interactions keep existed before HPV infection and thus impose momentous effect on persistence of stochastic HPV infection *via* VM-modulated host responses (Anahtar et al., 2015). In consistence, several studies indicated significant CVM differences between women with clearance of HPV infection and those with persistent infection (Di Paola et al., 2017; Berggrund et al., 2020; Liu et al., 2020; Mitra et al., 2020; Usyk et al., 2020). In addition, longitudinal studies indicated the vital impact of CVM on persistence of HPV infection (Mitra et al., 2020; Usyk et al., 2020). Therefore, clinical outcomes of HPV infection may be determined by CVM, which can function as a major immune modulator *via* microbe- and host-microbe interactions (Anahtar et al., 2015; Dai et al., 2021).

Bacterial vaginosis (BV)-associated anaerobes, such as *Gardnerella* and *Prevotella* which also accumulated for SIL-positive women in our study and other reports (Łaniewski et al., 2018; Chen et al., 2019; So et al., 2020), enabled persistent viral infection and subsequent disease development through impacting several cellular pathways (Holmes et al., 1985; Uren et al., 2005; Hedges et al., 2006; Cheriyan et al., 2009; Anderson et al., 2011). *Sneathia* spp., which also accumulated in CVM of BV patients, can promote carcinogenesis *via* activation of proinflammatory pathways and inhibition of immunocytotoxicity (Gur et al., 2015). This was consistent with previous reports, suggesting the positive association of BV with HPV infection and accumulation of vaginal obligate anaerobic bacteria in women with persistent HPV infection and cervical dysplasia progression (Liang et al., 2019). The above-mentioned information also explained the dramatically higher CVM diversity in HPV+HSIL group and higher ratio of NLD CVM for SIL-positive women in this study.

*Lactobacillus*-dominated CVM, which represented higher proportion in HPV- group in our study and other reports (Łaniewski et al., 2018; Chen et al., 2019; So et al., 2020), were mainly identified in women without genital infections. Vaginal *Lactobacillus* spp. can inhibit the colonization of common pathogens, like *Chlamydia trachomatis*, *Neisseria gonorrhoeae* and BV-associated *Gardnerella. vaginalis*, through produced lactic acid and bacteriocins (Dover et al., 2008; Graver and Wade, 2011; Gong et al., 2014; Mastromarino et al., 2014; Stoyancheva et al., 2014; Breshears et al., 2015; Kovachev, 2018). Additionally, *Lactobacillus* can prevent biofilm formation of pathogenic anaerobes *via* excreted biosurfactants and then inhibit overgrowth of those pathogens (Reid et al., 1999; Strus et al., 2006; Zárate and Nader-Macias, 2006; Ojala et al., 2014). *Prevotella* and *Gardnerella* had limited expression of H_2_O_2_-degrading enzymes and thus are inhibited by *Lactobacillus*-derived H_2_O_2_ (Vallor et al., 2001; Strus et al., 2006). Besides direct inhibition on pathogens, *Lactobacillus* also occupy niches to indirectly protect against pathogen colonization. For example, epithelium adhesin facilitates the adhesion of *L. crispatus* to genital mucosa and thus inhibits pilus-mediated adhesion of *G. vaginalis* (Ojala et al., 2014).

Though vaginal *Lactobacillus* play critical roles in protection against common pathogens which facilitated persistent HPV infection, not all *Lactobacillus*-dominated CVM benefit the host in the same manner. For instance, lactic acid has L- and D-isomer, the former is produced by *L. iners* and a variety of anaerobes, while the latter is mainly produced by *L. jensenii*, *L. crispatus*, *L. gasser* (Witkin et al., 2013). And *L. iners-*dominant CVM allows growth of strict anaerobes resulting in transition to non-*Lactobacillus*-dominant CVM, because *L. iners*-dominated CVM is less stable than other *Lactobacillus*-dominated CVM (Romero et al., 2014; Mitra et al., 2020). Conversely, *L. crispatus*-dominant CVM led to increased cervicovaginal mucus viscosity and promoted viral capture to prevent persistent infection (Nunn et al., 2015). Prior reports also found high frequency of *L. iners*-dominant CVM in women with persistent HPV infection and progression of cervical diseases (Oh et al., 2015; Seo et al., 2016; Di Paola et al., 2017; Zhang et al., 2018; So et al., 2020) as well as high frequency of *L. crispatus*-dominant CVM in women with natural clearance of HPV infection (Gajer et al., 2012; Romero et al., 2014; Mitra et al., 2020). Though we found significant CVM changes for women with HSIL, there were no conspicuous inter-group species-level *Lactobacillus* differences. This can be attributed to the role of non-*Lactobacillus* species in facilitating persistent HPV infection, such as unclassified OTUs, and to strain-specific *Lactobacillus* functions (Nunn et al., 2015; Lee et al., 2016; Liu et al., 2018; Schmid et al., 2018; Atassi et al., 2019; Al-Sadi et al., 2021). For example, *Lactobacillus acidophilus* strains induced different host responses *via* toll-like 2 receptors (Al-Sadi et al., 2021).

This study found significant CVM variation for women with HPV infection and HSIL *via* controlling confounding factors such as menopause and genital infections, but it should point out three main limitations. Firstly, this is a cross-sectional study and microbial samples in the same group were possibly heterogeneous. Based on follow-up investigation for the clinical outcomes of HPV-positive women, we could analyze the association of CVM with HPV infection more accurately. Secondly, there was no recorded nugent score, which may confounded our findings. Nonetheless, lack of those information should not impact our findings negatively in that our statistic analysis indicated the notable association of SIL level with CVM structure. Thirdly, 16S rDNA amplicon sequencing only analyzed the known bacterial components and there was no functional analysis for CVM. Given the important role of viral and fungal components in the CVM, cross-species RNA-Seq can be applied to unravel the complex host-microbe interactions and to explore functionally key microbial components in impacting the risk of persistent HPV infection.

To conclude, our study characterized CVM for women with different status of HPV infection and SIL progression, and found the significant CVM differences for women with HSIL. These findings provide additional evidence for the association of CVM with clinical outcomes of HPV infection for Chinese women. Additionally, this study partly supports the hypothesis that CVM should impact the risk of persistence of pre-existing HPV and SIL progression, instead of the risk of HPV acquisition.

## Data availability statement

The datasets presented in this study can be found in online repositories. The names of the repository/repositories and accession number(s) can be found below: https://db.cngb.org/search/project/CNP0002763/, CNP0002763.

## Ethics statement

This study was approved by the Ethics Committee of Peking University Shenzhen Hospital (registration number: 2021-006). The patients/participants provided their written informed consent to participate in this study.

## Author contributions

Conceptualization, RW, HD, and WD. Methodology, CG, JH, HC, DW, and WD. Software, QZ and WD. Formal analysis, QZ and WD. Resources, RW and HD. Data curation, SL. Writing—original draft preparation, WD and CG. Writing—review and editing, RW and HD. Visualization, WD. Supervision, RW and CL. Funding acquisition, WD and RW. All authors contributed to the article and approved the submitted version.

## Funding

This work was supported by Shenzhen High-level Hospital Construction Fund(grant number: YBH2019-260), Shenzhen Key Medical Discipline Construction Fund(grant number: No.SZXK027), Sanming Project of Medicine in Shenzhen (grant number: No.SZSM202011016) and Scientific Research Foundation of Peking University Shenzhen Hospital (grant number: No.KYQD2021075).

## Conflict of interest

The authors declare that the research was conducted in the absence of any commercial or financial relationships that could be construed as a potential conflict of interest.

## Publisher’s note

All claims expressed in this article are solely those of the authors and do not necessarily represent those of their affiliated organizations, or those of the publisher, the editors and the reviewers. Any product that may be evaluated in this article, or claim that may be made by its manufacturer, is not guaranteed or endorsed by the publisher.
